# Systematic Identification of Key Functional Modules and Genes in Gastric Cancer

**DOI:** 10.1155/2020/8853348

**Published:** 2020-11-16

**Authors:** Rui Wu, Jin-Yu Sun, Li-Li Zhao, Zhi-Ning Fan, Cheng Yang

**Affiliations:** ^1^Department of Digestive Endoscopy, The First Affiliated Hospital of Nanjing Medical University, 300 Guangzhou Road, 210029 Nanjing, Jiangsu, China; ^2^Department of Cardiology, The First Affiliated Hospital of Nanjing Medical University, 300 Guangzhou Road, 210029 Nanjing, Jiangsu, China; ^3^Department of Gastroenterology, The Affiliated Wuxi People's Hospital of Nanjing Medical University, 299 Qiangyang Road, 214023 Wuxi, Jiangsu, China

## Abstract

Gastric cancer (GC) is associated with high incidence and mortality rates worldwide. Differentially expressed gene (DEG) analysis and weighted gene coexpression network analysis (WGCNA) are important bioinformatic methods for screening core genes. In our study, DEG analysis and WGCNA were combined to screen the hub genes, and pathway enrichment analyses were performed on the DEGs. *SBNO2* was identified as the hub gene based on the intersection between the DEGs and the purple module in WGCNA. The expression and prognostic value of *SBNO2* were verified in UALCAN, GEPIA2, Human Cancer Metastasis Database, Kaplan–Meier plotter, and TIMER. We identified 1974 DEGs, and 28 modules were uncovered via WGCNA. The purple module was identified as the hub module in WGCNA. *SBNO2* was identified as the hub gene, which was upregulated in tumour tissues. Moreover, patients with GC and higher *SBNO2* expression had worse prognoses. In addition, *SBNO2* was suggested to play an important role in immune cell infiltration. In summary, based on DEGs and key modules related to GC, we identified *SBNO2* as a hub gene, thereby offering novel insights into the development and treatment of GC.

## 1. Introduction

Gastric cancer (GC) is associated with high incidence and mortality rates worldwide, especially in China, Japan, and Korea [1]. Annually, more than 1 million new cases of GC are diagnosed globally [2], including approximately 679,000 new cases (477,000 males, 202,000 females) in China in 2015 [3]. The stage of GC significantly determines the prognosis of patients. However, because of the occult and atypical symptoms of early GC, more than 60% of patients present with advanced disease at the time of diagnosis [4].

Although gastroscopy has greatly improved the detection of early GC, its use remains low. Despite the availability of a national GC screening program in Korea, only 56.3% of people were screened via gastroscopy in 2015, and people with severe disabilities had a markedly lower screening rate (51.9%) [5]. Meanwhile, compared with the large population in China, professionals and facilities dedicated to gastroscopy are relatively sparse [6]. Therefore, it is necessary to explore simpler, safer, and more efficient biological markers for the clinical diagnosis and prognostic assessment of patients with GC.

*SBNO2* was found to be expressed mainly in the spleen and bone marrow. It has been reported to play an important role in the development of Peutz–Jeghers syndrome, an autosomal-dominant hereditary disease with hamartomatous polyps of the gastrointestinal tract that carries a higher risk of gastrointestinal tumours [7, 8]. Meanwhile, *SBNO2* is one of the susceptibility loci of Crohn's disease and ulcerative colitis [9]. In addition, *SBNO2* is also linked to increased risks of cardiovascular disease and type 2 diabetes in conjunction with increasing body weight [10, 11].

In previous studies, most researchers focused on differentially expressed genes (DEGs). However, weighted gene coexpression network analysis (WGCNA) is increasingly applied to explore the relationships among genes across microarray or RNA sequence data, making it an effective method for screening hub genes [12]. In our study, integrated bioinformatic analysis was used to screen the core gene and verify its value in GC and prognosis surveillance. The combination of DEG analysis and WGCNA to screen hub genes can be beneficial for understanding the potential molecular mechanism of oncogenesis and tumour development. Our study may provide new insights into the clinical diagnosis and prognostic assessment of GC.

## 2. Methods

### 2.1. Data Acquisition and Preprocessing

The expression profile of GSE54129 was downloaded from Gene Expression Omnibus (GEO, https://www.ncbi.nlm.nih.gov/geo/), which provides comprehensive data on gene profiling and sequencing as an online database. GSE54129 contains 111 human GC tissues and 21 noncancerous gastric tissues, which were analysed via high-density oligonucleotide microarray. Later, the gene symbols were matched with probes after removing redundant data (e.g., time and null value), and the “limma” package in R software 3.4.1 was used to correct background, normalise quantiles, and summarise quantiles.

### 2.2. Identification of DEGs

The “limma” package in Bioconductor (http://www.bioconductor.org/) was applied to explore the DEGs between normal and gastric tumour tissues. The standard of adjusted *P* < 0.01 and ∣log_2_foldchange | >1 was set for significant DEGs according to the normalised gene expression levels.

### 2.3. Pathway Enrichment Analyses

Gene Ontology (GO) is a common method for annotating genes and their underlying biological phenomena. The ontology covers three domains: biological process, cellular component, and molecular function. The Kyoto Encyclopedia of Genes and Genomes (KEGG) is an integrated database resource for the large-scale molecular datasets generated via genome sequencing and other high-throughput experimental technologies [13]. The significant GO terms and pathways were identified using Fisher's exact test [14], and the adjusted *P* value was obtained using the Benjamini and Hochberg false discovery rate algorithm. GO and KEGG pathway analyses were performed on the DEGs using the “clusterProfiler” package in R. Furthermore, GOCluster analysis [15] was performed to generate a circular dendrogram of the data clustering via the default Euclidean distance and average linkage.

### 2.4. Establishment of WGCNA and Identification of Modules

The coexpression network was constructed using the freely accessible “WGCNA” package in R via the one-step network construction and module detection function. First, gene and sample data were imported into R software, and obvious outliers were removed. Second, the coexpression network was constructed via the automatic network construction function, and the soft-thresholding power of 9 was selected according to the scale-free topology criterion. Third, the hierarchical clustering dendrogram was applied to detect modules with different colors using minModuleSize and CutHeight values of 30 and 0.99, respectively. Fourth, the modules were correlated with clinical traits using module-trait associations, and genes were related to clinical traits based on module membership (MM) and gene significance (GS). Fifth, the connectivity of eigengenes in different modules was revealed via the topological overlap matrix method [16].

### 2.5. Module Preservation Evaluation

Zsummary is composed of four statistics related to density and three statistics related to connectivity [17]. As the value of Zsummary increases, the strength of evidence that the module is preserved in a certain condition/treatment becomes greater. However, Zsummary tends to increase with increasing module size. Therefore, when comparing the preservation statistics of modules with different sizes, it is important to observe the connectivity patterns among hundreds of nodes. In this case, medianRank can be used because it is based on the observed preservation statistics and is not affected by module size [18]. In our study, because the blue module contained far more genes than the purple module, medianRank was adopted. A module with a lower medianRank is more preserved than that with a higher medianRank.

### 2.6. Principal Component Analysis (PCA) and t-Distributed Stochastic Neighbour Embedding (t-SNE)

PCA was performed using “gmodels” and “scatterplot3d.” The genes in the purple module were examined to display the degree of overlap between samples in each of the normal and tumour samples. In addition, t-SNE was applied as a nonlinear dimensionality reduction method [19, 20], and it exhibited the ability to distinguish tumour tissue from normal tissue.

### 2.7. The Hub Genes Generated from DEGs and the Purple Module in WGCNA

A Venn diagram program (Supplementary Figure (available here)) was employed to reflect the intersection between DEGs and the purple module in WGCNA, which included 25 genes. Furthermore, the top 10 genes were screened as the hub genes based on GS, including *SBNO2*, *THRB*, *LOC102724788*, *BDH2*, *LIF*, *GNG12*, *KIAA0232*, *TEAD4*, *CXCL2*, and *RTEL*, in that order. Then, *SBNO2* was further explored in our study.

### 2.8. Protein-Protein Interaction (PPI) Network Construction

PPI pairs between *SBNO2* and its related genes were identified using the String database (https://string-db.org/) [21], and the PPI network was illustrated and visualised using Cytoscape software (version 3.5.0) [22, 23].

### 2.9. UALCAN, GEPIA2, and Human Cancer Metastasis Database (HCMDB) Analysis

*SBNO2* expression in GC and normal tissues was detected using the UALCAN (http://ualcan.path.uab.edu/) web portal, which is a user-friendly and interactive interface [24]. The expression data for *SBNO2* were obtained using the “Expression Analysis” module, and *P* was calculated. Furthermore, *SBNO2* expression was verified in the GEPIA2 database (http://gepia2.cancer-pku.cn/) [25] and HCMDB (http://hcmdb.i-sanger.com/index). HCMDB is an integrated database designed to analyse expression data and metastasis data of cancers collected from 124 previously published transcriptome datasets [26].

### 2.10. Kaplan–Meier (KM) Plotter Database Analysis

The online KM plotter (http://kmplot.com/analysis) database was employed to evaluate the prognostic impacts of *SBNO2* on overall survival (OS), and data for 876 patients with GC are contained in this database [27]. For KM analysis, all cases were ranked according to the expression level of *SBNO2* and then divided into two groups based on the median expression of *SBNO2*.

### 2.11. TIMER Database Analysis

The TIMER database (https://cistrome.shinyapps.io/timer/) [28] was used to analyse the association between *SBNO2* expression and the abundance of infiltrating immune cells, including B cells, CD8^+^ T cells, CD4^+^ T cells, macrophages, neutrophils, and dendritic cells. Meanwhile, the distributions of *SBNO2* expression levels in different cancers were also evaluated.

## 3. Results

### 3.1. DEG Identification and Pathway Enrichment Analyses

**Figure 1:**
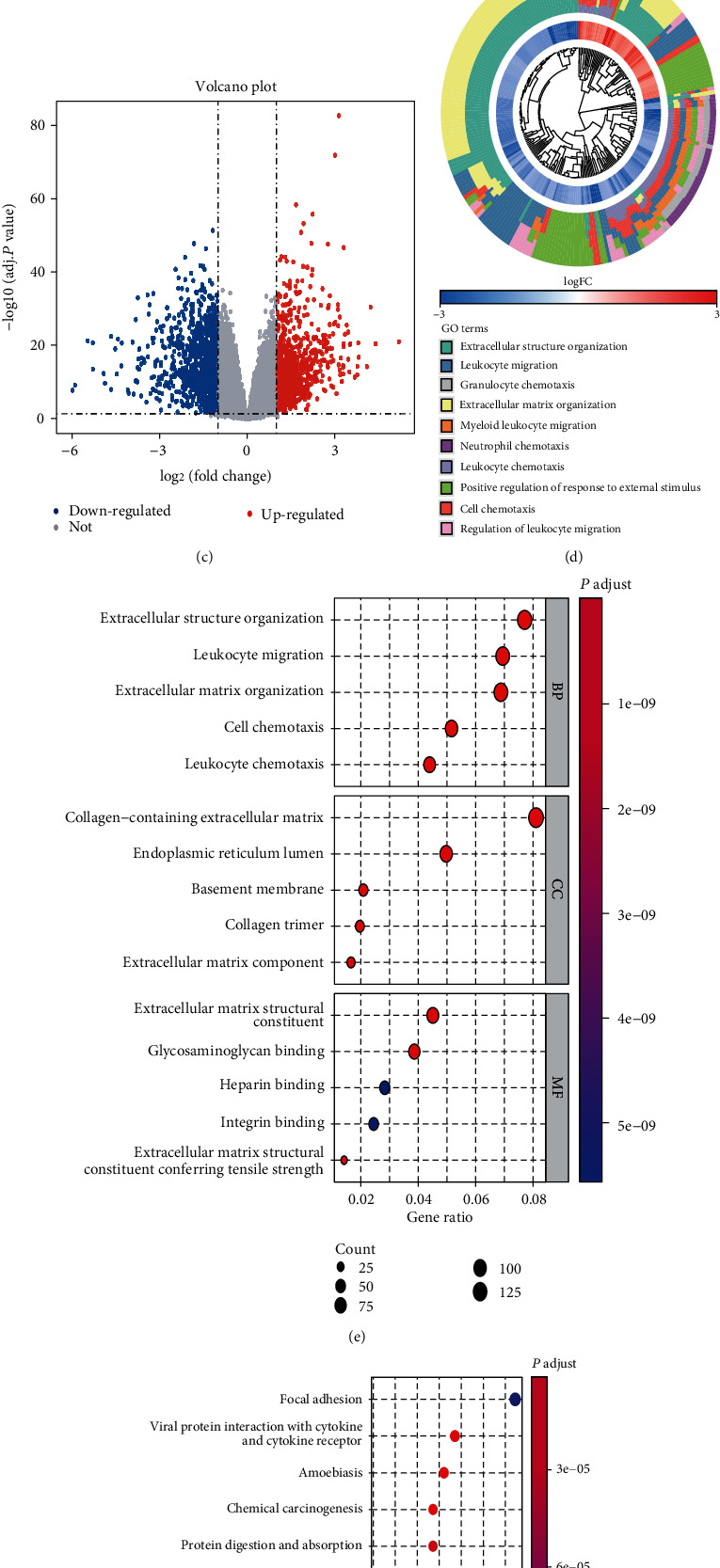
Identification of DEGs and pathway enrichment analyses. (a) Box plot of gene expression data before normalization of GSE54129. (b) Box plot of gene expression data after normalization of GSE54129. (c) Volcano plot of DEGs. (d) GOCluster analysis was performed to generate a circular dendrogram of the data clustering via default Euclidean distance and average linkage. (e) GO is used to annotate genes and their underlying biological phenomena. (f) KEGG is used for enrichment analyses of DEGs.

After preprocessing, 1974 DEGs were identified in GC tissues compared with their expression in normal tissues. As presented in the volcano plot, 1076 of these genes were upregulated in tumours, whereas 898 were downregulated (Figures 1(a)–1(c)). Meanwhile, GO analysis suggested that the DEGs may play important roles in extracellular structure organisation, leukocyte migration, granulocyte chemotaxis, extracellular matrix structural constituent, and other processes (Figures 1(d) and 1(e)). KEGG pathway enrichment of the DEGs was conducted, revealing that the focal adhesion, viral protein interaction with cytokine and cytokine receptor, and amoebiasis pathways were highly enriched in DEGs (Figure 1(f)).

### 3.2. WGCNA, PCA, and t-SNE

**Figure 2:**
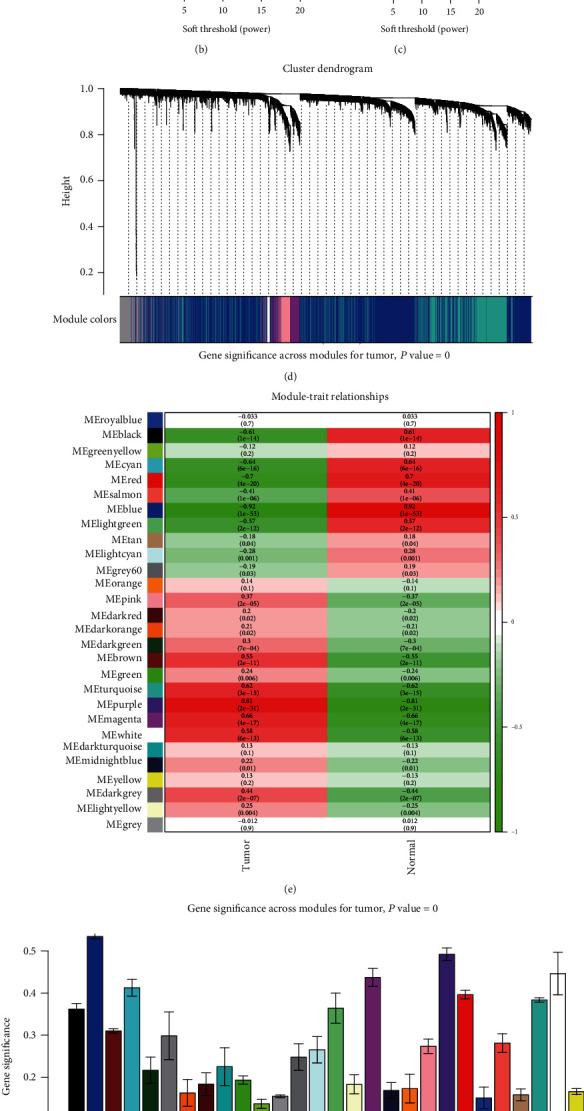
WGCNA analysis. (a) Sample dendrogram and trait heat map. (b) Analysis if the scale free fit index for various soft-thresholding powers (*β*). (c) Analysis of the mean connectivity of various soft-thresholding powers. (d) The cluster dendrogram of genes. Each branch in the figure represents one gene, and every color below represents one coexpression module. (e) Module-trait relationships. (f) Module significance of different modules. (g) Clustering of module eigengenes.

**Figure 3:**
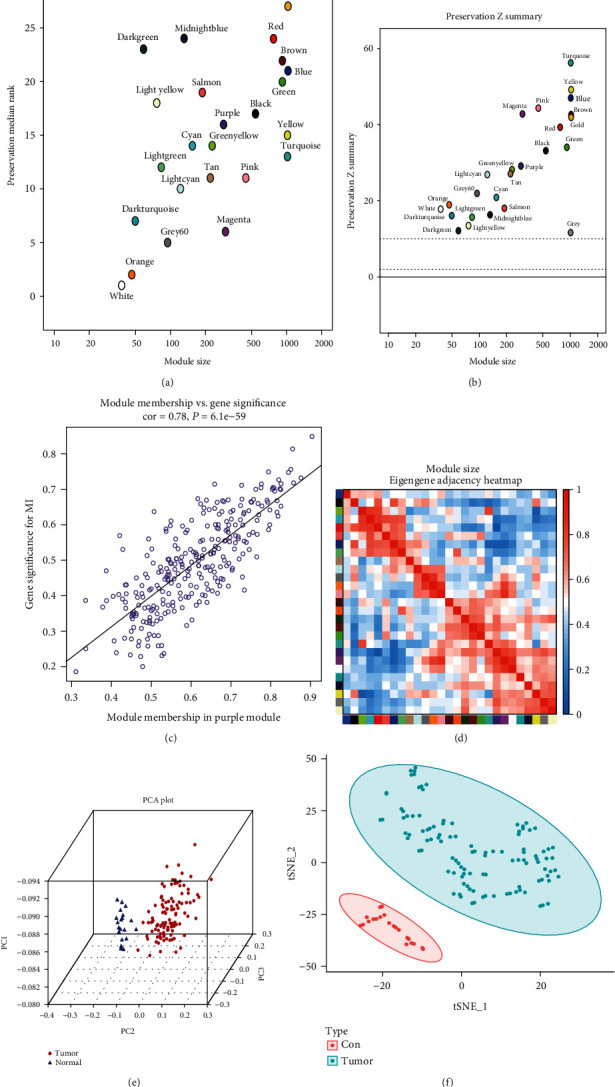
Screening of hub modules. (a) medianRank score analysis of different modules. (b) Zsummary score analysis of different modules. (c) The relationship between gene significance and module membership in the purple module. (d) Eigengene adjacency heat map. (e) The genes in the purple module show the degree of overlapping between samples in each of the normal and tumour by using PCA. (f) t-SNE was a nonlinear dimensionality reduction method to distinguish tumour from the normal tissues.

A gene coexpression network was constructed using a weighted expression correlation. The clustering was based on the expression data of GSE54129, which contains 111 human GC tissues and 21 normal gastric tissues (Figure 2(a)). The soft-thresholding power of 9 was set to ensure a scale-free network (Figures 2(b) and 2(c)). All 22,878 genes were assigned to 28 modules, which were associated with GC in WGCNA (Figure 2(d)). In total, 282 genes were assigned to the purple module, and 3620 genes were assigned to the blue module. These two modules were both significantly related to clinical traits (blue: correlationcoefficient = −0.92, *P* < 0.001; purple: correlationcoefficient = 0.81, *P* < 0.001; Figure 2(e)). Furthermore, the results of GS indicated that module significance (MS) was higher for the blue and purple modules than for the other modules (Figure 2(f)). However, the blue and purple modules were derived from different metamodules (branches) in the clustering of module eigengenes (Figure 2(g)). Because the blue module contained much higher number of genes than the purple module, Zsummary was not stable for comparing the blue and purple modules, and medianRank was adopted [18]. The result illustrated that the purple module had a lower medianRank, and it was identified as the key module (Figures 3(a) and 3(b)). The correlation coefficient between MM and GS was 0.78 (*P* < 0.001) for the purple module (Figure 3(c)). Furthermore, in eigengene adjacency heat map, the purple module was grouped together with the red module (Figure 3(d)). Additionally, the results of PCA and t-SNE both displayed satisfactory connectivity and the ability to distinguish purple module genes in response to tumour and normal tissues (Figures 3(e) and 3(f)).

### 3.3. The Overall Expression Levels and Prognostic Values of *SBNO2* in Patients with GC

**Figure 4:**
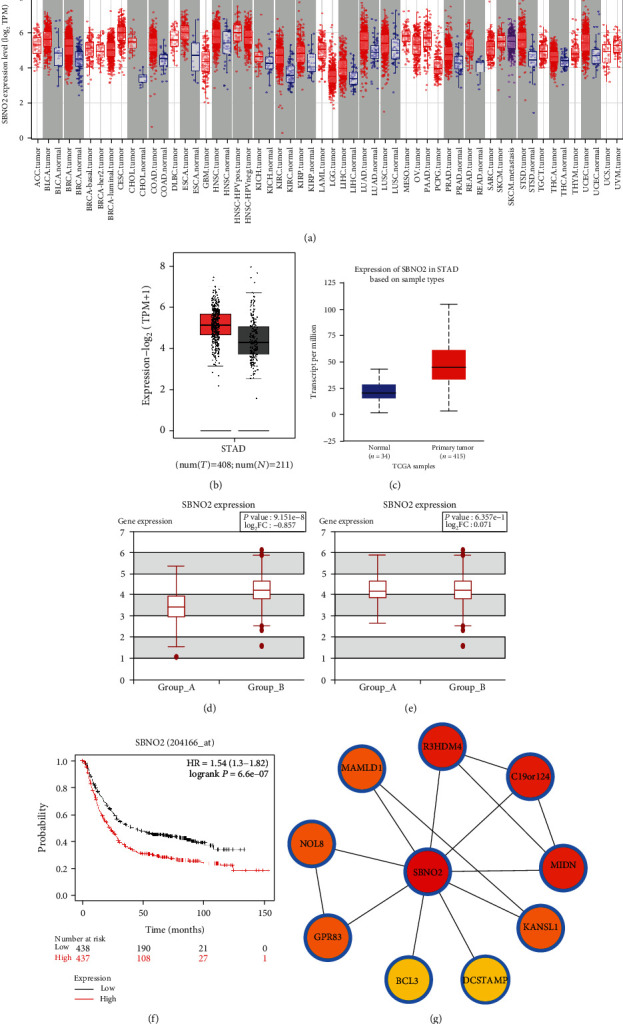
The overall expression levels and prognostic values of SBNO2. (a) The expression levels of SBNO2 in tumour and normal tissues were evaluated in the TIMER database. (b) The expression levels of SBNO2 were evaluated in the GEPIA2 database. (c) The expression levels of SBNO2 were evaluated in the UALCAN database. (d) The expression levels of SBNO2 were evaluated in the HCMDB database. (e) The expression levels of SBNO2 in metastatic and primary GC were evaluated in the HCMDB database. (f) The prognostic values of SBNO2 were evaluated. (g) PPI network was constructed.

We first evaluated *SBNO2* levels in tumour and normal tissues using TIMER and found that *SBNO2* expression was significantly elevated in GC (*P* < 0.001), as well as bladder urothelial carcinoma, cholangiocarcinoma, oesophageal carcinoma, and head and neck squamous cell carcinoma (Figure 4(a)). Then, GEPIA2, UALCAN, and HCMDB were used to verify the significantly higher expression of *SBNO2* in patients with GC, and the expected conclusion was obtained (all *P* < 0.001; Figures 4(b)–4(d)). However, there were no significant differences in *SBNO2* expression between patients with metastatic and primary GC (*P* > 0.05; Figure 4(e)). To assess the prognostic value of *SBNO2*, the KM curve was plotted. High *SBNO2* expression was notably associated with worse OS in 876 patients with GC (hazard ratio (HR) = 1.54, 95% confidence interval (CI) 1.30–1.82, *P* < 0.001; Figure 4(f)). In addition, the PPI network was analysed to further comprehend *SBNO2* and its relative genes (Figure 4(g)).

### 3.4. Subtype Analysis of Expression Levels and Prognostic Value of *SBNO2* in Patients with GC

**Figure 5:**
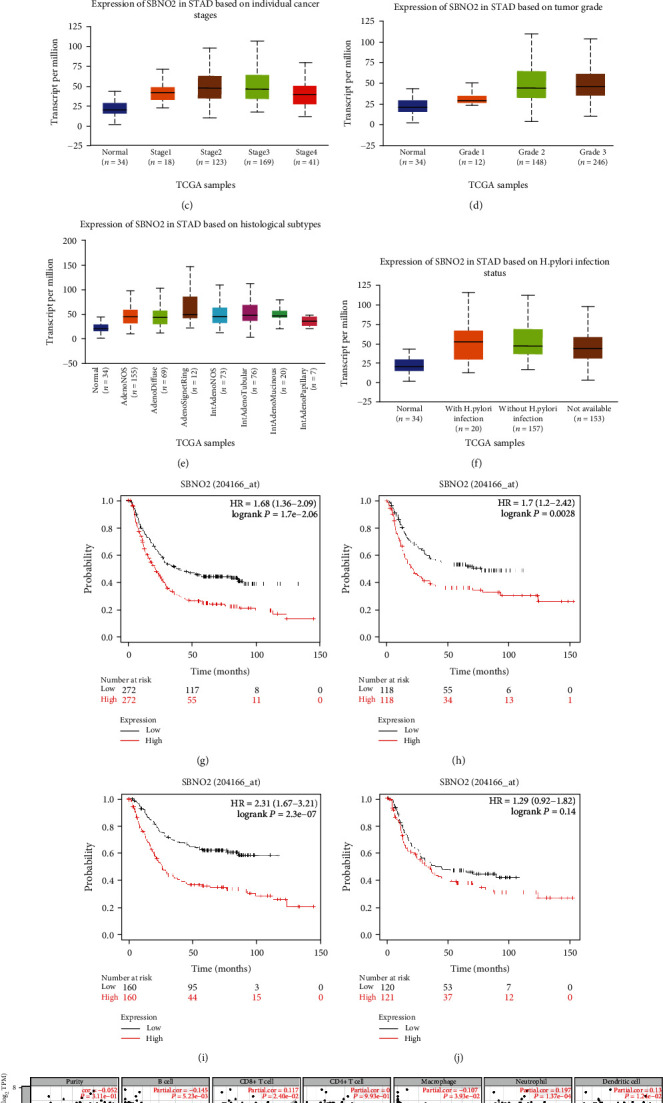
The subtype analysis of expression levels and prognostic values of SBNO2. (a–f) The different expressions of SBNO2 in gender, age, individual cancer stages, tumour grade, histological subtypes, and *H. pylori* infection status were explored in patients with GC. (g–j) Subtype analysis of prognostic values of SBNO2 was performed in gender and histological subtypes. (k) Immune cell infiltration of SBNO2 in patients with GC.

The differences in *SBNO2* expression according to gender, age, individual cancer stage, tumour grade, histological subtypes, and *Helicobacter pylori* infection status were explored in patients with GC. *SBNO2* mRNA expression was higher in men (*P* < 0.001) and women with GC (*P* < 0.001) than in healthy people (Figure 5(a)). *SBNO2* expression was higher in patients older than 40 years (*P* < 0.001; Figure 5(b)). Compared with the findings in normal tissues, *SBNO2* mRNA levels were higher in stage 1-4 GC (*P* < 0.001), and expression was the highest in stage 2 lesions (Figure 5(c)). *SBNO2* expression was higher in grade 1–3 GC (*P* < 0.001) than in normal tissues, and expression was the highest in grade 3 lesions (Figure 5(d)). *SBNO2* expression was higher in signet ring cell carcinoma than other GC pathological types (*P* < 0.001; Figure 5(e)). *SBNO2* expression was significantly higher in GC tissues than in normal tissues irrespective of *H. pylori* infection (*P* < 0.001; Figure 5(f)).

Furthermore, subtype analysis of the prognostic value of *SBNO2* was performed. Increased *SBNO2* mRNA expression was associated with poor OS in both men (HR = 1.68, 95%CI = 1.36–2.09, *P* < 0.001) and women with GC (HR = 1.70, 95%CI = 1.20–2.42, *P* = 0.003; Figures 5(g) and 5(h)). Similarly, subtype analysis of gastric pathology was performed, and increased *SBNO2* mRNA expression was associated with intestinal GC (HR = 2.31, 95%CI = 1.67–3.21, *P* < 0.001) but not with diffuse GC (HR = 1.29, 95%CI = 0.92–1.82, *P* = 0.14; Figures 5(i) and 5(j)).

### 3.5. Correlation of Immune Cell Infiltration and *SBNO2* in Patients with GC

Tumour-infiltrating lymphocytes have emerged as predictors of the sentinel lymph node status and survival in cancers [29]. Therefore, a comprehensive exploration of the correlation between *SBNO2* expression and immune cell infiltration was conducted using the TIMER database. *SBNO2* expression was negatively correlated with B cell infiltration (correlationcoefficient = −0.145, *P* < 0.05) and positively correlated with CD8^+^ T cell (correlationcoefficient = 0.117, *P* < 0.05) and dendritic cell infiltration (correlationcoefficient = 0.130, *P* < 0.05) (Figure 5(k)). These results strongly suggested that *SBNO2* plays an important role in regulating immune cell infiltration in GC.

## 4. Discussion

In our study, the expression profile of GSE54129 was analysed, and DEGs were identified in GC tissues in comparison with normal tissues. Moreover, the key GC-related pathways of the DEGs were analysed via GO and KEGG pathway analyses. Moreover, we used WGCNA to identify the core modules that were closely associated with GC. Furthermore, we identified 10 hub genes derived from the intersection between DEGs and the purple module in WGCNA, and the expression and prognostic value of *SBNO2* for GC were evaluated.

GO analysis suggested that DEGs play an important role in the extracellular structure organisation, leukocyte migration, granulocyte chemotaxis, and extracellular matrix structural constituent pathways, which has been proven by many studies [30–32]. KEGG pathway enrichment analysis of the DEGs was also conducted, and the focal adhesion, viral protein interaction with cytokine and cytokine receptor, and amoebiasis pathways were highly enriched in DEGs. The IL-17 signalling pathway also plays an important role in GC [33].

*SBNO2* was further explored in several terms. Through repeated verification in multiple databases, *SBNO2* is highly expressed in GC, and it has significant values in the follow-up of patients with GC. Takano et al. found that *SBNO* family genes included one *SBNO1* homologue and two *SBNO2* homologues (*SBNO2a* and *SBNO2b*) via whole-mount in situ hybridisation [34]. There is a conserved set of genes surrounding *SBNO2* in humans and other vertebrates, indicating an archetypal organisation within this region. They also reported that *SBNO2* is mainly expressed in blood cells and bone, whereas *SBNO1* is expressed in the developing brain. Furthermore, *SBNO2* has been reported to play an important role in the gastrointestinal system. *SBNO2* is one of the susceptibility loci of Crohn's disease, ulcerative colitis, and Peutz–Jeghers syndrome [7, 9], which may be closely related to many types of tumorigenesis. Meanwhile, Grill et al. reported that *SBNO2* is a novel inflammatory response factor. It is predominantly but not exclusively expressed by astrocytes in the central nervous system [35]. Our study further proved that *SBNO2* is associated with B cell, CD8^+^ T cell, and dendritic cell infiltration.

Furthermore, higher *SBNO2* expression is associated with BMI. The leucocyte epigenome-wide association study of 60 lean and 60 obese young women was performed using the Illumina Infinium HumanMethylation450 BeadChip [36], and *SBNO2* was found to be closely associated with obesity. Maruyama et al. [37] also reported that *SBNO2^−/−^* mice exhibited slightly lower body weight at 10 weeks of age than their wild-type counterparts. Recently, the SEER database was used to illustrate that among people older than 50 years, GC resection was associated with increased obesity over the period of 2002–2013 [38]. Meanwhile, Jang et al. [39] demonstrated that compared with people in the reference BMI range (22.6–25.0 kg/m^2^), those with higher BMI (>27.5 kg/m^2^) had an increased risk of GC (oddsratio = 1.48, 95%CI = 1.15–1.91). In our study, *SBNO2* was found to be closely related to GC, and higher *SBNO2* expression was liked to a higher risk of GC and worse prognosis. Therefore, we believe that *SBNO2* expression is higher in people with GC, and one possible cause of its increased expression is elevated BMI. Some studies reported that *SBNO2* increases the risk of cardiovascular disease and type 2 diabetes by increasing BMI [10, 11], but the mechanism by which *SBNO2* leads to GC has not yet been revealed. We believe that our research has provided new directions for exploring this issue.

## 5. Conclusions

Our research identified DEGs and key modules contributing to GC and clarified the expression levels and prognostic value of *SBNO2*, thereby offering novel insights into the development and treatment of gastric cancer.

## Data Availability

The datasets used and/or analyzed during the present study are available from the corresponding author on reasonable request.

## Supplementary Materials

Supplementary Materials Supplement Figure: the Venn diagram program was employed to reflect the intersection between DEGs and the purple module in WGCNA, which included 25 genes.
