# Supplementary figures and images for: Systematic Identification of Key Functional Modules and Genes in Gastric Cancer
Source: Biomed Res Int. 2020 Nov 16;2020:8853348. doi: 10.1155/2020/8853348 (PMC7685902; doi:10.1155/2020/8853348)

Purple module

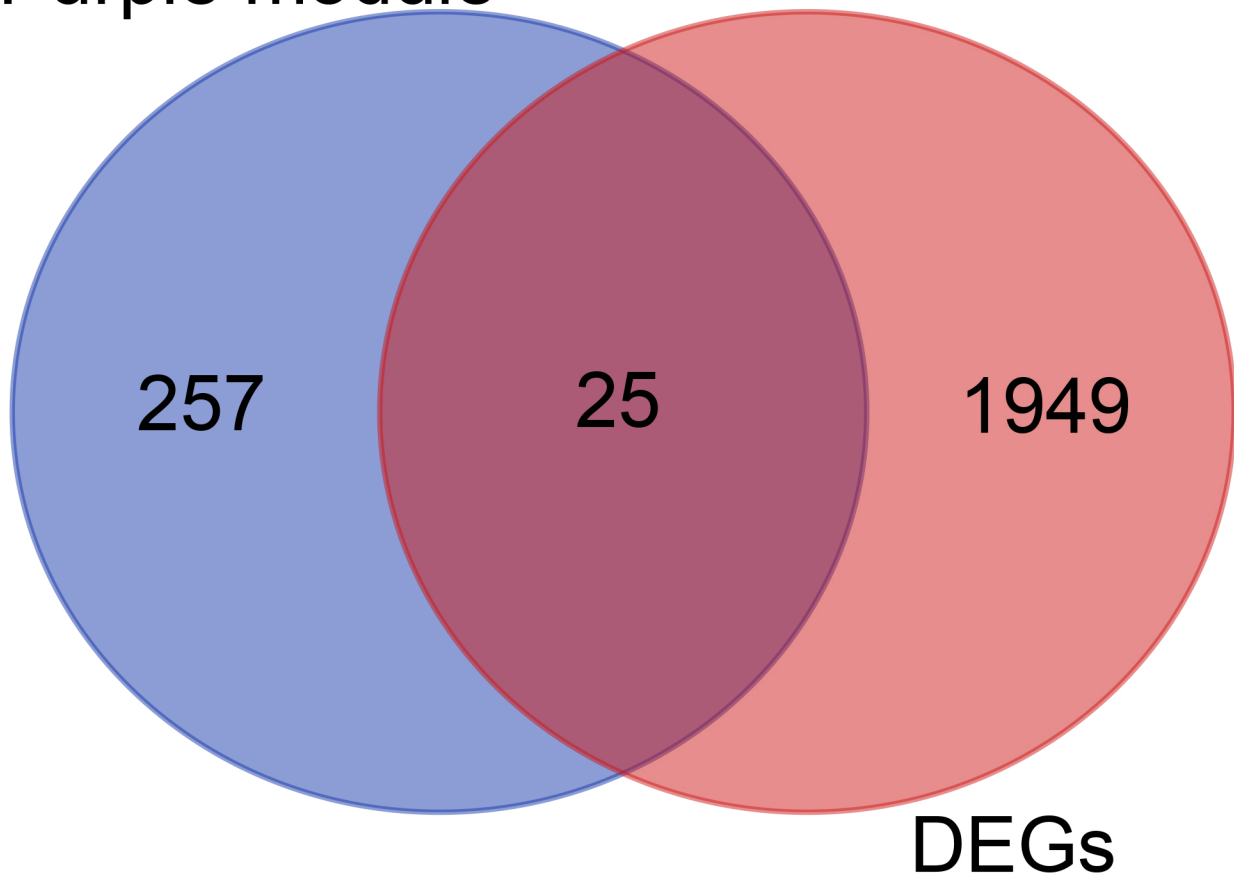

Supplement: Supplementary Materials — Supplement Figure: the Venn diagram program was employed to reflect the intersection between DEGs and the purple module in WGCNA, which included 25 genes. [file 8853348.f1.pdf]
